# Ethnomedicinal study of medicinal plants used by Mizo tribes in Champhai district of Mizoram, India

**DOI:** 10.1186/s13002-022-00520-0

**Published:** 2022-03-24

**Authors:** T. B. C. Laldingliani, Nurpen Meitei Thangjam, R. Zomuanawma, Laldingngheti Bawitlung, Anirban Pal, Awadhesh Kumar

**Affiliations:** 1 Department of Horticulture, Aromatic and Medicinal Plants, School of Earth Sciences and Natural Resources Management, Mizoram University, Aizawl, 796004 India; 2 Department of Botany, School of Life Science, Mizoram University, Aizawl, 796004 India; 3 Bioprospection and Product Development, CSIR-Central Institute of Medicinal and Aromatic Plants, CIMAP, Lucknow, 226015 India

**Keywords:** Ayurveda, Champhai, Ethnomedicinal, Indo-Burma hotspot, Tribal

## Abstract

**Background:**

Medicinal plants have been used countless times for curing diseases mainly in developing countries. They are easily available with little to no side effects when compared to modern medicine. This manuscript encompasses information on ethnomedicinal plants in Champhai district, located in the North East Region (NER) of India. The region lies within Indo-Burma biodiversity hotspot. This study will be the first quantitative report on the ethnomedicinal plants used by the local tribes of this region. Knowledge of medicinal plants is mostly acquired by word of mouth, and the knowledge is dying among the local youths with the prevalence of modern medicine. Hence, there is urgency in deciphering and recording such information.

**Methods:**

Information was gathered through interviews with 200 informants across 15 villages of the Champhai district. From the data obtained, we evaluate indices such as used report (UR), frequency of citation (FC), informant consensus factor (*F*_ic_), cultural values (CVs) and relative importance (RI) for all the plant species. Secondary data were obtained from scientific databases such as Pubmed, Sci Finder and Science Direct. The scientific name of the plants was matched and arranged in consultation with the working list of all plant species (http://www.theplantlist.org).

**Results:**

Totally, 93 plant species from 53 families and 85 genera were recorded. The most common families are Euphorbiaceae and Asteraceae with six and five species representatives, respectively. Leaves were the most frequently used part of a plant and were usually used in the form of decoction. *Curcuma longa* has the most cultural value (27.28 CVs) with the highest used report (136 FC), and the highest RI value was *Phyllanthus emblica*. The main illness categories as per Frequency of citation were muscle/bone problem (0.962 *F*_ic_), gastro-intestinal disease (0.956 *F*_ic_) and skin care (0.953 *F*_ic_).

**Conclusion:**

The people of Mizoram living in the Champhai district have an immense knowledge of ethnomedicinal plants. There were no side effects recorded for consuming ethnomedicinal plants. We observed that there is a scope of scientific validation of 10 plant species for their pharmacological activity and 13 species for the phytochemical characterisation or isolation of the phytochemicals. This might pave the path for developing a scientifically validated botanical or lead to semisyntheic derivatives intended for modern medicine.

## Background

Plants have been known to be a major source of diverse chemical compounds possessing both medicinal properties and commercial value. There have been several reports on medicinal plants as a source for drug discovery. However, new diseases will likely continue to emerge along with drug-resistant pathogens. This dynamic nature of pathogens has constantly challenged researchers to look for alternatives. The past few decades have witnessed the surge in ethnomedicinal plant research [1], one of the reasons being that the natural products have played an important role in the development of drugs, contributing more than 50% of clinical drugs in the pharmaceutical industry [2]. Further, the rapid growth in human population has raised the demand which in turn has increased the quest for novel plant resources, triggering a threat to natural resources [3].

Traditional knowledge and practices of herbal remedies have been passed on to new generations over the centuries and will continue to do so, with some variations taking place every generation. Plants have been the essential source for therapeutic regimes since ages, and traditional practices are proved to have little known side effects besides their low cost and easy availability. India has been well known worldwide for its indigenous traditional pieces of knowledge and practices from ancient times, through different systems of medicine such as Ayurveda, Siddha and Unani [4]. Although more than 427 tribal communities are having vast diversity of ancient traditions, still there has been criticism of ethnomedicines due to regional variation, political and socio-economic challenges [5]. Reports are stating that several plants have been increasingly utilised by the indigenous people of India [1]. Generally, in India, it was estimated that 6,000 species are used in traditional and herbal medicine which represent about 75% of the needs of the third world, and meanwhile, 3,000 plants were officially acknowledged due to their medicinal values [6].

The healthcare system of India witnesses a wide variation encompassing urban and rural populations which rely on both modern and traditional systems of medicine. The recently implemented Ayushman Bharat Pradhan Mantri Jan Arogya Yojana from the Commonwealth Fund enables cashless secondary and tertiary care at private facilities [7]. Besides, health insurance schemes also exist for institutions and factories. Catering to the huge population has its limitations, and thus, many of the ailments are treated either by traditional healers or through traditional knowledge and practices, especially in remote areas. One such state in the North-Eastern part of India is Mizoram.

Although some researchers [8–13] have documented and identified several ethnomedicinal plants of Mizoram mentioning their mode of preparation, usage, distribution and habitat, they mostly reported from the core areas of the cities. Their studies highlighted the qualitative data. However, there are no in-depth ethnobotanical studies recorded in Champhai district. Therefore, the present study aims to carry out a quantitative study using different cultural importance indices to assess the most valued plants and document the ethnomedicinal practices involving medicinal plants of the Champhai district of Mizoram, India. Their practical knowledge has been established based on more than a century of credence and observation.

## Methods

### Description of the study area

Mizoram lies within the Indo-Burma biodiversity hotspot region and shares two international borders with Bangladesh in the west and Myanmar in the east. According to Champion and Seth (1968), Mizoram forests are classified into Tropical semi-evergreen forests, tropical wet evergreen forests and mountain sub-tropical pine forests [14]. The study area, i.e. the Champhai district, is classified as a rural area where healthcare facilities are relatively poor which drives the people to rely on traditional medicines. The traditional healers using medicinal plant-based formulations for various ailments indicate that traditional medicines are still one of the mainstays in their contemporary health care. It is felt that prospection and research on the medicinal plants that play such an important role in the health care of Mizo tribes need a more intensified effort.

**Fig. 1:**
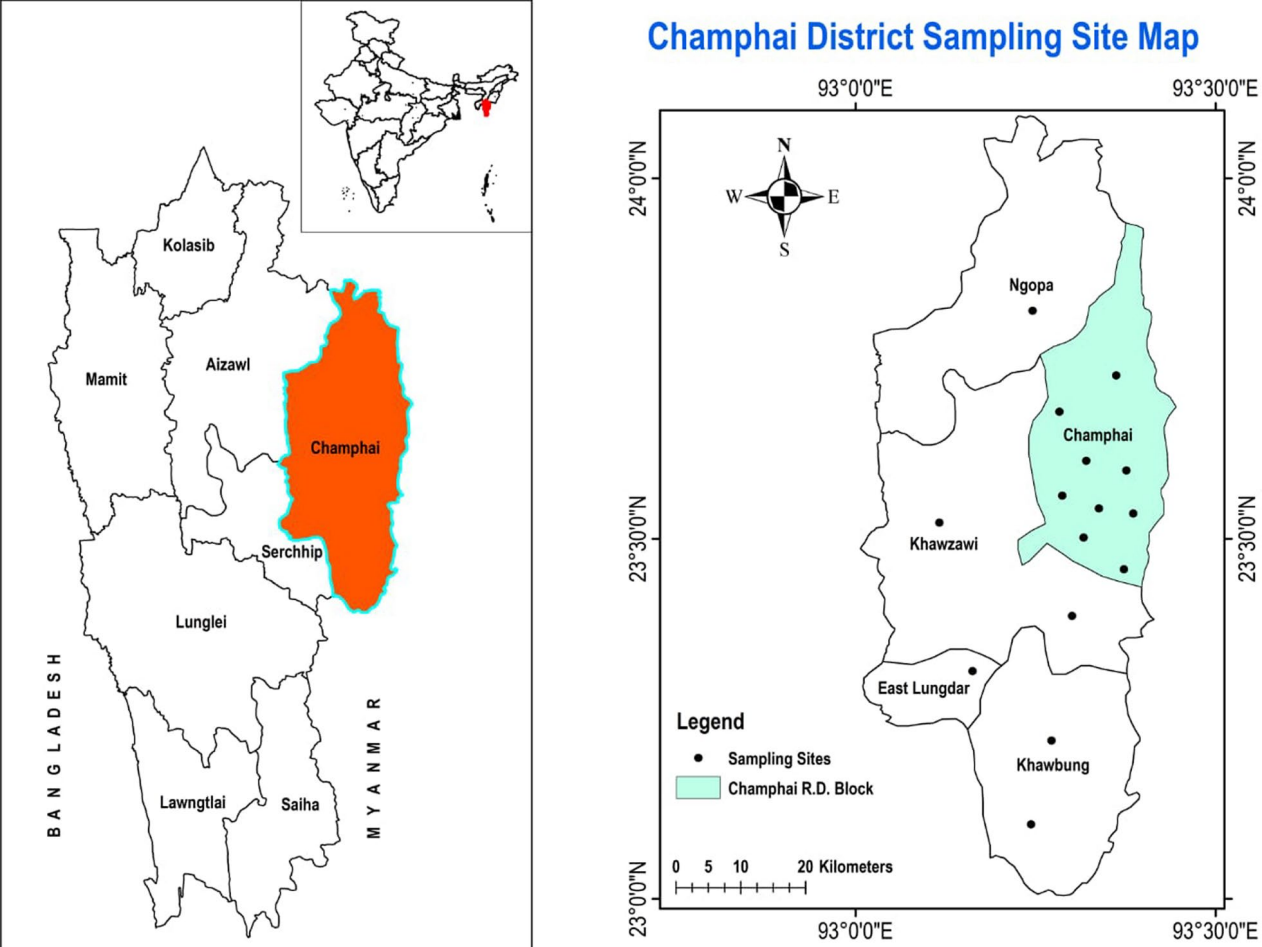
Location of the present study area: Champhai district, Mizoram, India

Champhai is one of the 8 districts in Mizoram, amidst the North-East Region of India. It is located in the eastern part of Mizoram, internationally bordered by Myanmar and therefore becoming the main gate of trading for India and Myanmar. It lies between 23.456° N latitude and 93.328°E longitude. The average annual rainfall is approximately 1814 mm, and the temperature remains around 18.6 °C which is slightly colder than the rest of the state during winter. The total land area is 3185.83 sq kilometres at an elevation around 1678 m above sea level, population density is 10 per sq kilometres (32,734). According to an official Census (2011), Champhai reported a population of 1,26,000, of which male and female were 62,357 and 63,388, respectively [15]. The study area was divided into 15 village council areas (Vengthlang, Vengthlang North, Venglai, Vengsang, Electric veng, Kanan, Kahrawt, Bethel, New Champhai, Zotlang, Hmunhmeltha, Tualcheng, Ngopa, Khawzawl and East Lungdar) for extensive data collection (Fig. 1). The majority of people living in this area are Mizo tribe and use the Mizo dialect in common.

### Investigative method

In the field study, formal questionnaires were distributed to each participant while having face to face interviews at their residence. At least 16 people were interviewed in each village council area. Only those people who have knowledge in the art of preparing medicines either for their families or their neighbourhood were considered for the interaction. The interactions primarily focussed on their experience, type of dosage form, duration of usage, any adverse effects observed and the source of their knowledge about the plant and their parts used. This information was then correlated with the scientific data curated from related databases (Pubmed, SciFinder and Science Direct). In most of the cases, the voucher specimens were deposited (Herbarium, Mizoram University, Aizawl, Mizoram, India) for their authentication and archiving.

### Characteristics of demographic data

This demonstrated the socio-economic information of the informant including qualities like age, sex, education level and occupation. Using random sampling method, 200 people (12–14 individuals from each village) in the ages group of 18–71 years were interviewed, of which 112 and 82 were males and females, respectively. Respondents belonged to various professions while some were students. Most of the informants do not engage in full-time ethnomedicinal practice or as a profession. The feature of demographical characteristics obtained in the study is tabulated below (Table 1).

**Table 1:** Demographic characteristics of informants (*n* = 200)

Age	Number	In percent (%)
*Demographic characteristics*		
18–30	42	21
31–50	69	34.5
51–70	76	38
71 above	13	6.5
*Sex*		
Male	112	56
Female	88	44
*Educational level*		
Primary	30	15
Middle	44	22
High School	56	28
Higher Secondary	32	16
University	38	19
*Occupation*		
Self Employed (farmer, carpenter, bussiness)	70	35
Govt. Employed (teacher, bank, officer)	65	32.5
Unemployed Student, housewives)	65	32.5

### Quantitative analysis

#### Frequency of citation

Frequency of citation was used to further examine the primary data by finding the sum of total citations/usage reports for a particular species. The usage report is the quotation of one plant by an informant [16].

#### Use value

Use value or UV is used to express the correlative importance of each particular plant species locally known and was calculated by the following equation [17].

$${\text{UV}} = \sum \frac{{U_{i} }}{n}$$

where ‘*U*_*i*_’ represents the number of citations of each species by the informants and ‘*n*’ represents the total number of informants in the study area. The larger the number of citations, the greater is the use-value.

#### Informant consensus factor

*F*_ic_ or ICF is used to represent the consistency of the information among the informants, indicating whether there were shared knowledge and concurrence in the use of plants for treating the ailment category among the plant’s users in the study area. It was calculated by the following equation [18].

$$F_{{{\text{ic}}}} = \frac{{{\text{N}}_{{{\text{ur}}}} - N_{t} }}{{{\text{N}}_{{{\text{ur}}}} - 1}}$$

where ‘*N*_ur_’ refers to the number of users reports in each illness category and ‘*N*_*t*_’ refers to the number of plant species used for a particular illness category by all the informants.

Further, *F*_ic_ value with 1 or either close to 1 indicates that a large number of informants had agreed on using few plants for curing an illness category while low *F*_ic_ value signified that there was an argument on using medicinal plants to treat illness amidst the category.

#### Relative importance

When calculating RI, both the informants who mentioned the useful plant species and their various kinds of uses are considered. So, it was calculated by the following equation [19].

$${\text{RI}} = {\text{NUC}} + {\text{NT}}$$

where ‘NUC’ refers to the number of illnesses use category of each species divided by the total number of most use categories among the species and ‘NT’ refers to the number of illness types of uses of each species divided by the total number of most types of uses among the species.

#### Cultural values

In this index, the use category is taken into account and it was calculated by using the following equation [20].

$${\text{CVs}} = {\text{UCs}} \times {\text{ICs}} \times \sum {\text{IUCs}}$$

where ‘UCs’ is the number of the used reports for each species divided by the total number of use categories of that species. ‘ICs’ is the number of informants who mention each plant as effective divided by the total number of informants, and ∑IUCs is the number of informants who report the use of each species divided by the total number of informants.

## Results

### Demographic characteristics

All the 200 respondents were randomly selected from 15 village council areas interviewing at least 16 persons in each area with no equal separation of male–female ratio. Amongst them, the elderly in their seventies and above occupied 6.5% only, while people between 31 and 50 years old occupied 34.5%. The average age among the informants was 54 years. Mizoram is the second most literate state in the country (2011 census), and all the informants were literate having at least primary school level education. Out of the total informants, 32.5% were engaged in government jobs like teachers, officers, while 35% were self-employed like farmers, carpenters, skilled workers, small businesses and the rest 32.5% of the informants were unemployed including students and housewives (Table 1).

### Taxonomy identification

In the present study, 93 medicinal plant species belonging to 53 families and 85 genera have been reported for treating various kinds of ailments. The most prominent families were Euphorbiaceae with 6 plant species followed by Asteraceae with 5 plant species and 4 species each among Cucurbitaceae and Zingiberaceae. Liliaceae, Fabaceae, Verbenaceae, Solanaceae, Rutaceae, Anacardiaceae are with 3 species each while Orchidaceae, Combrethaceae Theaceae, Arecaceae, Apocynaceae, Musaceae, Rubiaceae, Scrophulariaceae, Lamiaceace, Mimosaceae, Smilacaceae are with 2 species each and other 34 families with one species each as shown in Table 2. The high usage report of this large family like Euphorbiaceae (6 species), Asteraceae (5 species) and Zingiberaceae (4 species) occupied 10.8%, 9.2% and 8.35% of the total used report, respectively, indicating that most people in the study area are inclined to use plants that are easily available and abundant around them (Table 2).

**Table 2:** Name of plant families with number of species and used report

Family	No of species	No of used report	%
Euphorbiaceae	6	294	10.8
Asteraceae	5	250	9.2
Cucurbitaceae	4	82	3.02
Zingiberaceae	4	227	8.35
Liliaceae	3	76	2.8
Fabaceae	3	56	2.06
Verbenaceae	3	78	2.87
Solanaceae	3	83	3.05
Rutaceae	3	120	4.42
Musaceae	3	80	2.94
Anacardiaceae	3	79	2.91
Apocynaceae	2	53	1.95
Orchidaceae	2	14	0.52
Combretaceae	2	55	1.66
Meliaceae	2	54	1.99
Theaceae	2	28	1.03
Arecaceae	2	86	3.17
Rubiaceae	2	38	1.4
Scrophulariaceae	2	48	1.77
Lamiaceae	2	40	1.47
Mimosaceae	2	53	1.95
Smilacaceae	2	26	0.96
Bromeliaceae	1	49	1.8
Begoniaceae	1	37	1.36
Amaranthaceae	1	15	0.55
Betulaceae	1	7	0.26
Cannabinaceae	1	36	1.32
Apiaceae	1	29	1.07
Caricaceae	1	53	1.95
Fagaceae	1	17	0.63
Araceae	1	2	0.07
Dilleniaceae	1	22	0.81
Discoreaceae	1	27	0.99
Caryophyllaceae	1	28	1.03
Eleagnaceae	1	31	1.14
Proteaceae	1	15	0.55
Malvaceae	1	4	0.15
Saururaceae	1	16	0.59
Convulvulaceae	1	37	1.36
Rosaceae	1	14	0.52
Campanulaceae	1	25	0.92
Myrsinaceae	1	4	0.15
Magnoliaceae	1	18	0.66
Clusiaceae	1	18	0.66
Moraceae	1	2	0.07
Bignoliaceae	1	37	1.36
Pandanaceae	1	24	0.88
Phyllanthaceae	1	48	1.77
Plantaginaceae	1	36	1.32
Myrtaceae	1	98	3.61
Polypodiaceae	1	12	0.44
Punicaceae	1	31	1.14
Poaceae	1	15	0.55

### Frequency of usage of parts of plants

**Fig. 2:**
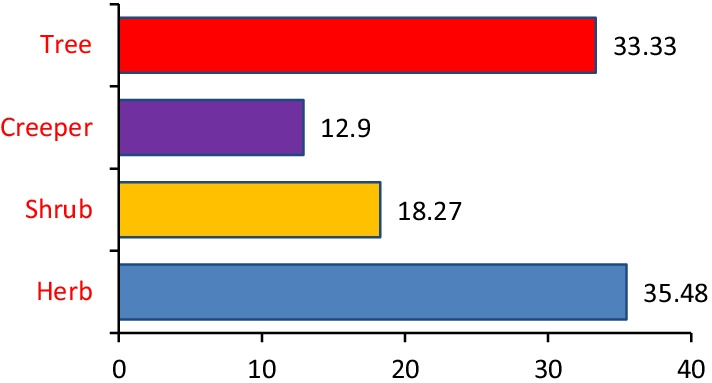
Percentage of plants habit

**Fig. 3:**
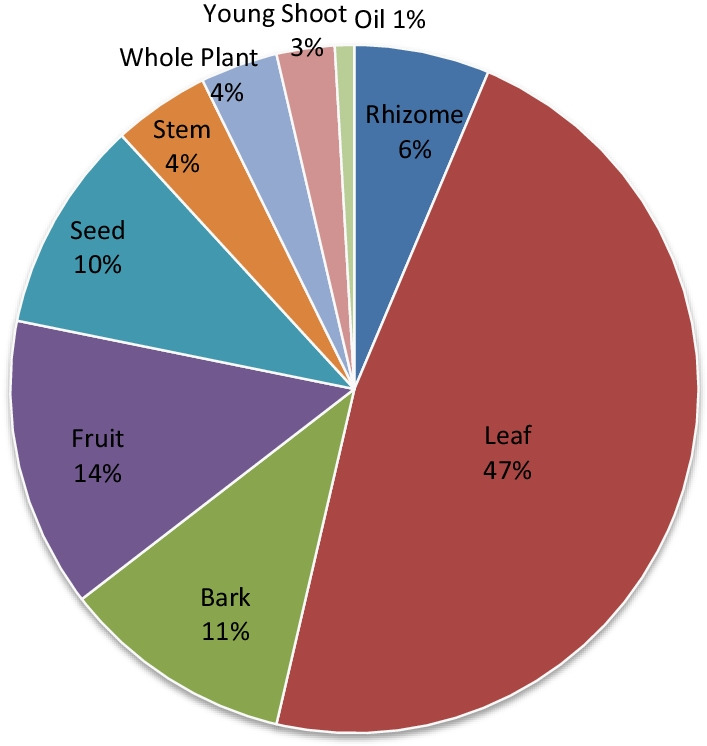
Percentage of parts used

The most commonly used medicinal plants fell under herbs (35.5%) followed by trees (33.3%), shurbs (18.3%) and creepers (12.9%) as shown in (Fig. 2). Among the parts, leaves, fruits and barks were mainly utilised by the informants (Fig. 3). A detailed analysis concluded that leaves (47%) followed by fruits (14%), barks (11%), seeds (10%), rhizomes (6%), stems (4%), young shoot (2%), oil (1%) and in some cases the whole plant (3%) were used for ethnomedicinal purposes.

### Mode of preparation and administration

**Fig. 4:**
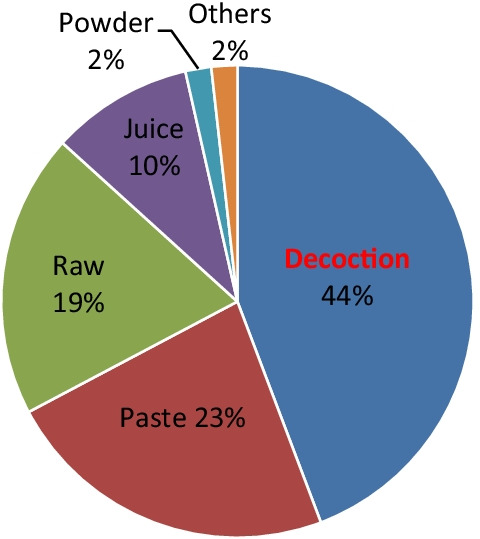
Distribution of formulation usage

The mode of formulation preparation or administration was observed to be in the form of decoction (44.2%) followed by paste (23%), raw (19.5%), juice (9.73%), powder (1.77%) and others like maceration and oil (1.77%) (Fig. 4.).

### Usage analysis based on the treatment of ailments

The total number of user reports documented in this study was 2717, in which all different illnesses were categorised into 16 groups using International Classification of Primary Care (ICPC) with a slight modification. Among the illness category, the gastro-intestinal disease has the highest usage report (940) followed by skincare (259) cardiovascular (222), kidney disease (196), hyperglycaemia (175), ENT (159), genito-urinary disease (139) and so on as shown in Table 3.

**Table 3:** Usage analysis with illness category and their term

Illness categories	Medical term	Local term	Frequency of usage
Dental Care (DC)	Tooth decay Toothpaste	Hamuat/Hanget Hanawhna	57
Skin Care (SC)	Pimple, burn, Face pack, boil, chickenpox, Measles, Herpes	Bawl, Kang, Sentut, Khawihli, Awmvel, Tangseh Hmai chiahna,	259
Hair Care (HC)	Growth enhances, shining, hair fall	Sam thatna, Sam tletna, Sam tla	16
Eyes/Nose/Ears/Mouth (ENT)	Otorrhoea, eye itching, Sinusitis, Tonsilitis	Beng kherh, Mit thak, Sinus, Tonsil	159
Genito-urinary Disease (GUD)	Delivery pain, placenta discharge, urine retention	Nau neih zawh hlam tlakna / na, Zun in	139
Kidney Disease (KD)	Nephrolithiasis, kidney failure	Kal a lungte awm, Kalna, Kal a hnai awm	196
High glucose level (HGL)	Diabetes type I & II	Zunthlum	175
Cancer Disease (CD)	Breast cancer, Leukaemia, lung cancer, etc.	Hnute Cancer, Thisen cancer, Chuap cancer leh dangte	75
Liver Problem (LP)	Jaundice, Hepatitis B & C, cholelithiasis, Malaria	Thinlian, Hepatitis, Malaria, Mit a lungte awm	100
Cardiovascular Problem (CP)	Hypertension, Heart problem	Bp sang leh hniam, lung thalo	222
Muscular/Bone Problem (MBP)	Rheumatoid, Arthritis, Sciatica	Ruh seh/Sehpui, thana, Scaitica	81
Respiratory System illness (RSI)	Cold, Cough, Asthma, lung disease	Hritlang, Awmna, Khuh, Thawhah	42
Gastro-intestinal Disease (GID)	Ulcer, stomach pain, Dysentry, Diarrhoea, Digestion, Hemorrhoid, Constipation, Intestinal worms, internal bleeding	Ulcer, Pumna, Ek khal, Pile na, Puar nuamlo, Khawthalo, Santen, Rul hlut, Internal bleeding	940
Wound healing (WH)	Inflammation	Pem thar /Pilh damdawi, Pan, vung	126
Poisonous Bites (PB)	Snake bite, Scorpion bite, dog bite, Wasp sting	Rul chuk, Ui seh, Khuai zuk, Khawmual kaikuang seh	45
General Health (GH)	Fever, Headache, cold, Immuno-booster, Energy booster, etc	Luna, Khawsik, Taksa chakna, Hriselna	81
		Total	2717

### Data analysis

#### Frequency of citation

Among the total number of user reports (UR) cited, *Curcuma longa* L. (136 FC), *Flueggea virosa* (Roxb. ex Willd.) Royle (126 FC), *Psidium guajava* L. (98 FC), *Chromolaena odorata* (L.) R.M. King & H. Rob. (87 FC), *Mikania micrantha* Kunth. (82 FC), *Citrus limon* (L.) Osbeck (68 FC), *Carica papaya* L. (53 FC), *Ananas comosus* (L.) Merr. (49 FC), *Sarcococca pruniformis* Lindl. (49 FC), *Phyllanthus emblica* L. (48 FC), *Rhus chinensis* Mill. (45 FC), *Clerodendrum glandulosum* Lindl. (44 FC), *Senecio scandens* Buch- Ham. ex D. Don (43 FC) were those species having the highest FC (Table 4).

**Table 4:** List of plant species with their use value (UV)

Sl. no	Species name; voucher no	Family name	Local name	^a^Habit	^b^Part Use	Mode of administration	Ailments & UR	FC	UV	Isolated chemical compounds	Bioactivity
1	*Allium cepa* L. (HAMP20045)	Liliaceae	Purun sen	H	Rt (Bulb)	Raw	Hair care (12), headache (1)	13	0.065	S-alk(en)yl-substituted cysteine sulphoxides, quercetin, kaempferol [21]	Antioxidant, appetiser Antimicrobial, Hypertension, hair regrowth [22, 23]
2	*Allium sativum L.* (HAMP20046)	Liliaceae	Purun var	H	Rt (Bulb)	Raw	Tension (10), toothache (2), cold (12), pimple (7)	31	0.155	S-allyl-cysteine, Allyl thiosulfinate (Allicin), S-ally-mercapto cysteine [24, 25]	Fever, hypertension, antitumor, antioxidant, antimicrobial, tooth disease, immune boost, etc. [26–28]
3	*Aloe vera* (L.) Burm.f. (HAMP20047)	Liliaceae	Rul lei	H	Lf	Raw	Burn (21), Stomachache (2), skin care (9)	32	0.16	Vitamin E, Sulfuric acid, butyl heptadecylic ester, 1-Tetradecyne [29]	Antimicrobial, anti- inflammatory, laxative, antidiarrhoea, wound healing, antiaging, antioxidant, etc. [30, 31]
4	*Alstonia scholaris* (L.) R. Br. (HAMP20006)	Apocynaceae	Thuamriat	T	Br	Decoction	Ulcer (15)	15	0.075	Alstonine, picrinine, akuammicine, echitamine [32]	Antimicrobial, anti-asthmatic, antioxidant, anti-ulcers, anticancer, rheumatic, inflammatory, wound healing, etc. [33, 34]
5	*Ananas comosus* (L.) Merr. (HAMP20019)	Bromeliaceae	Lakhuihthei	Sh	Lf, Fr	Raw, paste	Ulcer (12), seizure (1), Hypertension (10), urinary infection (16), lung disease (10)	49	0.245	1-O-feruloylglycerol, tricin, 2, 4-dichlorobenzoic acid, etc. [35]	Anti-inflammatory, anti-thrombotic, antioxidant, antiedematous, anthelminthic, diuretic, rheumatoid, anticancer, antimicrobial, etc. [36, 37]
6	*Anoectochilus brevilabris* Lindl. (HAMP20062)	Orchidaceae	Hnah mawi	H	Lf	Paste	Pile problem (12)	12	0.06	Not reported	Not reported
7	*Anogeissus acuminata* (Roxb. ex DC.) Wall. Ex Guillem. &Perr. (HAMP20023)	Combretaceae	Zairum	T	Br	Decoction,	Ulcer (26)	26	0.13	Castamollilin, Grandinin, (-)-Secoisolariciresinol, 2,3-bis-(4-Hydroxybenzyl butadiene [38]	Hypoglycemic, wound healing, cytoxicity, antibacterial, etc. [39, 40]
8	*Aporosa octandra* (Buch-Ham. ex D. Don) Vickery (HAMP20035)	Euphorbiaceae	Chhawntual	T	Lf, Br	Decoction	Ulcer (5), uterus problem (11)	16	0.08	2-Methyl-3-en-butyl-cyclohexyl Phthalate, (R)-Coclaurine (AO-5) [41]	Antioxidant, anthelmintic, oxidative stress, D-galactose induce protectivity, etc. [42, 43]
9	*Azadirachta indica* A. Juss (HAMP20052)	Meliaceae	Neem	T	Lf	Decoction	Malaria (16), jaundice (8)	24	0.12	Diepoxyazadirol, flowerine, flowerone, O-methylazadir-onolide [44]	Immunostimulant, antiviral, analgesic, anti-inflammatory, antiulcer, antioxidant, antimicrobial, hepatoprotective, antimalarial, antipyretic, hypoglycemic, etc. [45]
10	*Begonia inflata* C.B. Clark. (HAMP20016)	Begoniaceae	Sekhupthur	H, Cr	Lf	Paste, juice	Pile problem (12), diarrhoea (11), dysentery (14)	37	0.185	Not reported	Not reported
11	*Benincasa hispida* (Thunb.) Cogn. (HAMP20028)	Cucurbitaceae	Maipawl	H, Cr	Fr	Decoction	Stomach problem (4), digestion (10), diarrhoea (15)	29	0.145	Pentanoic acid, 5-hydroxy-2,4- dibutyl phenyl esters, Palmitic acid, 9,12-Octadeca-dienylchloride [46]	Anti-obesity; anti-ulcer; anti-inflammatory; antioxidant; anti-diarrhoeal; anxiolytic; antidiabetic, antinociceptive, etc. [47, 48]
12	*Beta vulgaris* L. (HAMP20001)	Amaranthaceae	Beet root	H	Rh	Raw, juice	Aneamia (3), immuno- booster (8), cancer (4)	15	0.075	Apigenin, Luteolin, Isoscutellarein 7-O-glucosyl, 8-O-xylosid, Caffeoyl-6-(3,4-dihydroxy benzoyl) β-D-glucoside [49]	Antioxidant, anticancer, hepatoprotective, anti-inflammatory, antimicrobial, anti-hypertension, hypoglycemic [50, 51]
13	*Betula alnoides* Buch-Ham. ex D. Don (HAMP20017)	Betulaceae	Hriang	T	Lf	Paste	Tooth paste (7)	7	0.035	α-pinene; a-terpineol; limonene; camphor; β-pinene [52]	Cytotoxicity, anti-inflammatory, antioxidant, antimicrobial, etc. [2, 53]
14	*Bischofia javanica* Blume (HAMP20036)	Euphorbiaceae	Khuang thli	T	Lf	Paste	Toothache (13)	13	0.065	3,4-dihydroxyphenylethyl alcohol, isotachioside, catechin, epicatechin, gallocatechin [54]	Antimicrobial, antiulcer, cytoxicity, antidysentry, anthelminthic, etc. [55, 56]
15	*Cajanus cajan* (L.) Millsp. (HAMP20041)	Fabaceae	Behliang	Sh	Lf	Decoction	Jaundice (25), intestinal worms (3)	28	0.14	3,5-bis1,1-dimethylethyl, Tetradecanoic acid, allyl hexadecyl, ester, n-Hexadecanoic acid [57]	Antimicrobial, antidiabetic, antioxidant, glycemic, anthelmintic, hepatoprotective, etc. [58]
16	*Callicarpa arborea* Roxb. (HAMP20087)	Verbenaceae	Hnah kiah	T	Lf, Br	Decoction	Stomach ache (9), diabetes (16), convulsion (7)	32	0.16	Martynoside, Isomartynoside, Ursolic acid, Antiarol rutinoside [59]	Antioxidant, antimicrobial, analgesic, anti-inflammatory, neuroprotective, antidiabetic, etc. [59]
17	*Camellia sinensis* (L.) Kuntze (HAMP20082)	Theaceae	Thingpuife	Sh	Lf	Raw, Decoction	Toothace (7), itchy eye (7)	14	0.07	Caffeine, Theobromine, Gallic acid, Ampelopsin, epicatechin-3-O-gallate, catechin-3-O-gallate [60]	Antimicrobial, tooth decay, antioxidant, anticancer, anti-obesity, antidiabetic, anti-inflammatory, etc. [61, 62]
18	*Cannabis sativa* L. (HAMP20021)	Cannabinaceae	Trip/ Kanza	H	Lf	Raw,	Stomach ache (12), diarrhoea (24)	36	0.18	Cannabigerol, cannabichromene, cannabidiol, cannabisativa, delta 9-Tetrahydrocannabinol [63]	Antioxidant, anti-allergic, anti-inflammatory, analgesic, anti-tumour, anti-diabetic, antibiotic [64]
19	*Centella asiatica* (L.) Urb. (HAMP20005)	Apiaceae	Darbengbur /lambak	H	WP	Decoction	Stomach ache (8), urinary infection (17), kidney disease (2), eye pain (2)	29	0.145	Centellin, asiaticin, centellicin [65]	Ati-ulcer, neuroprotective, antidepressant, anticonvulsion, immunostimulating, antioxidant, antibacterial, kidney injury protectivity, etc. [66, 67]
20	*Capsicum annuum* L. (HAMP20084)	Solanaceae	Hmarhcha	H	Fr	Paste	Wasp sting (9), burn (3), toothache (4), boil (2)	18	0.09	Ascorbic acids, quercetin, luteolin, chrysoeriol, hydroxycinnamic acids [68]	Anti-inflammatory, antimicrobial, antiviral, anticancer, analgesic, etc. [69]
21	*Carica papaya* L. (HAMP20026)	Caricaceae	Thingfanghma	T	Lf, Fr sap	Paste, raw	Tonsil (1), face pack (3), cancer (16), diabetes (11), dog bite (17), milk booster in mother (5)	53	0.265	Benzyl-β-d glucoside, β-sistosterol, β-sistosterol, oleic acids [70]	Antimalaria, antidiuretic, antimicrobial, anthelminthic, hepatoprotective, immunomodulatory, etc. [71, 72]
22	*Castanopsis tribuloides* (Sm.) A. DC. (HAMP20044)	Fagaceae	Thingsia	T	Br	Paste	Toothache (17)	17	0.085	Not reported	Antioxidant [73]
23	*Catharanthus roseus* (L.) G. Don (HAMP20007)	Apocynaceae	Kumtluang	T	Lf	Decoction	Hypertension (18), diabetes (20)	38	0.19	4-O-caffeoylquinic acid, quercetin-3- O-(6-O-rhamnosyl-glucoside), kaempferol-3-O- (6-O-rhamnosyl-glucoside) [74]	Antihypercholesterollemi, antideuretic, antibacterial, antimalaria, antiviral, cytotoxic, antidiabetic, antihyperglycemic antidiarrhoeal, antioxidant, anthelminthic, etc. [75, 76]
24	*Cheilocostus speciosus* (J. Koenig) C.D. Specht (HAMP20090)	Zingiberaceae	Sumbul	Sh	Rh	Decoction	Kidney disease (5), urinary problem (16), stomach ache (18)	39	0.195	24-hydroxytriacontan-26-one, sitosterol, cyloartanol, methyl triacontanoate [77]	Antifungal, insecticidal, antioxidant [78, 79]
25	*Chromolaena odorata* (L.) R.M. King & H. Rob. (HAMP20011)	Asteraceae	Tlangsam	H	Lf	Decoction, raw	Kidney disease (18), diarrhoea (13), stomach, ache (8), jaundice (14), wound (34)	87	0.435	α & β-pinenes,1,8-Cineole, β-Calacorene, β-Copaen-4α-ol [80]	Antipyretic, analgesic, anti-inflammatory, antispasmodiac, antimalaria, antioxidant, antimicrobial, etc. [81, 82]
26	*Citrus aurantiifolia* (Christm.) Swingle (HAMP20074)	Rutaceae	Champara	T	Fr	Juice	Stomach problem (7), digestion (9)	16	0.08	Pinene, Sabinene limonene, Myrcene, Telinene [83]	Antimicrobial, antioxidant, antiobesity, anthelminthic [84, 85]
27	*Citrus maxima* (Burm.) Merr. (HAMP20075)	Rutaceae	Sertawk	Sh	S	Raw	Hypertension (36)	36	0.18	Naringenin,5,7-dihydroxylcoumarin, 1, 3,5-trihydroxyhenzene, xanthotoxol [86]	Antimicrobial, analgesic, antioxidant, anti-obesity, anti-inflammatory, etc. [87, 88]
28	*Citrus limon* (L.) Osbeck (HAMP20076)	Rutaceae	Nimbu	T	Fr	Juice	Stomach problem (24), digestion (44)	68	0.34	Ascorbic acid, y-Aminobutyric acid, alanine, aspartic acid, arginine [89]	Anticancer, antiparasitic anti-inflammatory, antimicrobial, [90]
29	*Clerodendrum glandulosum* Lindl. (HAMP20088)	Verbenaceae	Phuihnam	Sh	Lf	Decoction	Hypertension (44)	44	0.22	Strongyloster, lupeol, n hentriacontane, palmitic acid, 2-pentadecyn-1-ol, hexacosane, vitamin E [91]	2-pentadecyn-1-ol, hexacosane, vitamin E [91]
30	*Colocasia esculenta* (L.) Schott (HAMP20008)	Araceae	Dawl	H	St sap	Juice	Vaginal discharge/ Lochia (2)	2	0.01	14α-methyl-5α-cholesta-9, 24-diene-3b, 7α-diol; 9, 12, cyanidin 3-glucoside; 9, 12, 13-trihydroxy-(E)- 10-octadecenoic acid [92]	Antihepatotoxicity, anthelminthic, anticancer, antimicrobial, antioxidant, anti-inflammatory, etc. [92–95]
31	*Combretum wallichii* DC (HAMP20024)	Combretaceae	Leihruisen	Sh	Sh	Raw	Tonsil (19)	19	0.095	Not reported	Anthelminthic [96]
32	*Crassocephalum crepidioides* (Benth.) S. Moore (HAMP20012)	Asteraceae	Buar thau	H	Lf	Paste	Wound bleeding (14)	14	0.07	(E)-β-farnesene, α-humulene, cis-β-guaiene, α-bulnesene [97]	Anti-tumour, cytoxicity, antidiabetic [98, 99]
33	*Cucurbita maxima* Duchesne (HAMP20029)	Cucurbitaceae	Mai	H, Cr	S	Raw	Instestinl worm (15)	15	0.075	Oleic acid, linoleic acid, palmitic acid, caffeic, syringic, vanillic, p-coumaric [100]	Antidiabetic, anticancer, antiobesity, antihelmintic, cytotoxicity, antibacterial, anti-inflammatory, anti-parasitic [101]
34	*Curcuma caesia* Roxb. (HAMP20091)	Zingiberaceae	Ailaidum	H	Rh	Raw, juice	Stomach ache (4), diarrhoea (18)	22	0.11	α, β-pineneeucalyptol, camphor, camphene, gallic acid, quercetin [102]	Antimicrobial, analgesic, antioxidant, antiulcer, antimutagenic, anti-asthmatic, anthelminthic, etc. [103]
35	*Curcuma longa* L. (HAMP20092)	Zingiberaceae	Aieng	H	Rh	Powder, juice	Ulcer (67), diarrhoea (4), derma care (30), stomach ache (35)	136	0.68	Curcumin, ar-turmerone, β-sesquiphellandrene, curcumenol [104]	Antimicrobial, anticancer gastrointestinal and respiratory disorder, anti-inflammatory, anti-diabetic, anti-allergic, hepatoprotective, anti-dermatophytic, neuroprotective, etc. [105]
36	Cucumis sativus L.h (HAMP20030)	Cucurbitaceae	Fanghma	H, Cr	Lf	Decoction, raw	Malaria (8), derma care (17)	25	0.125	Myristic acid, karounidiol, avenasterol, palmitoleic acid, alpha-linolenic acid [106]	Antimicrobial, antiulcer, antitumour, wound healing, hypoglycemic, hypolipidemic, anti-inflammatory, antioxidant, etc. [107]
37	*Dichrocephala integrifolia* (L.f.) Kuntze (HAMP20013)	Asteraceae	Vawkek tumtual	H	Lf	Decoction	Kidney disease (24)	24	0.12	Stearic acid, stigmasta-7,22-dien-3-ol, epifriedelanol, Methyl stearate, tritetracontane [108]	Antimicrobial, cytotoxity, antidiarhoeal, antioxidant, anti-inflammatory, neuroprotectivity [109–111]
38	*Dillenia pentagyna* Roxb. (HAMP20032)	Dilleniaceae	Kaihzawl	T	Lf, Br	Decoction	Diarrhoea (21), kidney disease (1)	22	0.11	Dillenetin, betunaldehyde, betulinic acid, quercetin, kaempferol glucoside, lupeol [112]	Antimicrobial, antiviral antioxidant, anticancer, antidiabetic, anti-inflammatory [112]
39	*Dioscorea alata* L. (HAMP20033)	Discoreaceae	Bachhim	H, Cr	Fr	Decoction	Cancer (27)	27	0.135	Hydro-Q9 chromene, γ-tocopherol-9, 1-feruloylglycerol, RRR-α-tocopherol [113]	Antimicrobial, antioxidant, anti-inflammatory, antidiabetic, etc. [114, 115]
40	*Drymaria cordata* (L.) Willd. ex Schult. (HAMP20027)	Caryophyllaceae	Changkal rit	H, Cr	Lf	Decoction, paste	Rheumatism (24), dysentery (4)	28	0.14	Stigmasterol, cerebroside [116]	Analgesic, anxiolytic, antipyretic, antitussive, antibacterial, anti-inflammatory [116]
41	*Dysoxylum excelsum* Blume. (HAMP20051)	Meliaceae	Thingthupui	T	YS	Decoction	Diarrhoea (18), hyper tension (12)	30	0.15	Isodauc-6-ene-10β,14-diol; 4-epi-isodauc-6-ene-10β,14-diol; 4-epi-6α,10β-dihydroxy-artabotrol [117]	Not reported
42	*Elaeagnus caudata* Schltdl. ex Momiy. (HAMP20034)	Elaegnaceae	Sarzuk	T	Lf	Decoction	Vaginal discharge/ Lochia (31)	31	0.155	Not reported	Expelling placenta, miscarriage, jaundice [118, 119]
43	*Elaeis guineensis* Jacq. (HAMP20009)	Arecaceae	Oil palm	T	Oil	Oil	Wound (1), burn (1), hair care (3)	5	0.025	3-isobutyl-2-methoxypyrazine; acetoin; 2-acetyl-1-pyrroline; ethyl hexanoate; 3-methylbutyl xacetate [120]	Antioxidant, wound healing, antimicrobial, anti-inflammatory, cardiovascular effect, antidiabetic, anticancer, etc. [121, 122]
44	*Embelia vestita* Roxb. (HAMP20010)	Arecaceae	Tling	Sh	Lf	Decoction	Measles (16), chickenpox (65)	81	0.405	Not reported	Not reported
45	*Ensete glaucum* (Roxb.) Cheesman (HAMP20057)	Musaceae	Saisu	H	St	Decoction	Nephrolithiasis (34)	34	0.17	Not reported	Not reported
46	*Ensete superbum* (Roxb.) Cheesman (HAMP20058)	Musaceae	Changel/ Tumbu	H	St sap, Fr	Juice	Snake bite (6), kidney disease (1), diabetes (4), WBC deficiency (23)	34	0.17	Pentadecanoic acid; 4H-Pyran-4-one, 2,3-dihydro-3, 5-dihydroxy-6-methyl-; 2-Furancarboxaldehyde, 5-(hydroxymethyl) [123]	Kidney stone preventivity, antioxidant, etc. [124]
47	*Erythrina stricta* Roxb. (HAMP20042)	Fabaceae	Fartuah	T	Br	Decoction	Ulcer (8), kidney disease (2)	10	0.05	β-caryophyllene; δ-cadenine; alpinum isoflavone; obovatin; isovanillin [125]	Antimicrobial, epilepsy, antioxidant, leprosy, anti-inflammatory, etc. [126, 127]
48	*Eulophia nuda* Lindl. (HAMP20062)	Orchidaceae	Nauban	H	WP	Decoction	Diarrhoea (2)	2	0.01	Eulophiol;3,4-dihydroxy-3,5,5- trimethoxybibenzyl; nudol; lupeol [128]	Antimicrobial, anti-inflammatory, cytotoxic, antioxidant, anticancer, antiasthmatic/ antibronchitis [128, 129]
49	*Euphorbia milii* Des Moul. (HAMP20037)	Euphorbiaceae	Hlinglukhum	H	Lf	Raw	Diarrhoea (52)	52	0.26	Abruquinone B; eremopetasitenin A; ellagic acid; isopetasoside; 7,8-Dihydroxycoumarin; [130]	Antimicrobial, antioxidant, cytoxicity, antinociceptive, anthelmintic, etc. [131, 132]
50	*Euphorbia royleana* Boiss. (HAMP20038)	Euphorbiaceae	Chawng	Sh	Lf sap	Juice	Otorrhoea (38)	38	0.19	Antiquorine A; Eurifoloid D; sandaracopimaradienolal; 15-isopimaradien-18-a [133]	Antitumour, anti-arthritic, antimicrobial, antioxidant, cytotoxicity, anti-inflammatory, etc. [134, 135]
51	*Flueggea virosa* (Roxb. ex Willd.) Royle (HAMP20039)	Euphorbiaceae	Saisiak	Sh	Lf	Decoction	Diabetes (59), stomach ache (17), chicken pox (50)	126	0.63	11-O-acetyl bergenin; gallic acid; virosecurinine; kaempferol; β-sitosterol; quercetin [136]	Anti-inflammatory, anti-pyretic, anti-hepatitis C, etc. [137, 138]
52	*Gomphogyne cissiformis* Griff. (HAMP20031)	Cucurbitaceae	Lalruanga dawi bur	H, Cr	Fr	Juice	Hypertension (6), diabetes (7)	13	0.065	Not reported	Not reported
53	*Hedyotis scandens* Roxb. (HAMP20072)	Rubiaceae	Kelhnam tur	H	Lf	Decoction	Kidney disease (4), pain relief (7), diabetes (10)	21	0.105	Hedyotoside A; hedyotoside B; hedyotoside C, D & E [139]	Antimicrobial, abdominal pain [140, 141]
54	*Helicia robusta* (Roxb.) R.Br. ex Blume (HAMP20069)	Proteaceae	Pasal-taka-za	T	Lf	Decoction	Placenta discharge (6), stomach pain (8), kidney disorder (1)	15	0.075	Not reported	Not reported
55	*Hibiscus sinensis* Mill. (HAMP20052)	Malvaceae	Midum par	Sh	Lf	Paste	Boil (4)	4	0.02	Quercetin-3,5-diglucoside, undecanoic acid, cyanidin chorides, rachidic acid, cyanin [142]	Antioxidant, antipyretic, antimicrobial, antifertility, anticonvulsive, etc. [142, 143]
56	*Houttuynia cordata* Thunb. (HAMP20077)	Saururaceae	Ui thinthang	H	Lf	Juice	Sainus problem (16)	16	0.08	Quercetin-3-O-β-D-galactoside-7-O-β-D-glucoside, aristolactam A & B, β-Sitosterol, N-phenethyl-benzamide [144]	Antiviral, antitumour, antioxidant, anti-inflammatory, antimicrobial, anti-SARS, immunomodulator, etc. [144]
57	*Ipomoea batatas* (L.) Lam. (HAMP20025)	Convovulaceae	Kawl-ba-hra	H, Cr	YS	Raw	Digestion (37)	37	0.185	3-mono-O-caffeoylquinic acid; caffeic acid; vitamin C; kaempferol [145] [145]	Antidiabetic, anticancer, antioxidant, antiulcer, cardiovascular effect [145]
58	*Lablab purpureus* (L.) Sweet (HAMP20043)	Fabaceae	Bepui	Sh	Lf	Paste	Vaccine pain relief (18)	18	0.09	Phytic acid, linoleic acid, linolenic acid [146]	Antidiabetic, anti-inflammatory, analgesic, cytotoxicity, antimicrobial, antioxidant, hypolipidemic [147]
59	*Laurocerasus undulata* (Buch-Ham. ex D. Don) M. Roem. (HAMP20071)	Rosaceae	Thei-ar-lung	H	Lf	Decoction	Heart disease (14)	14	0.07	Not reported	Not reported
60	*Lindernia ruellioides* (Colsm.) Pennell (HAMP20078)	Scrophulariaceae	Thasuih	H	WP	Paste	Sciatica (24)	24	0.12	Linderruelliosides A & B; plantainoside A; acteoside, desrhamnosylverbascoside [148]	Anti-inflammatory, antitumour, antiulcer, analgesic [149]
61	*Lobelia angulata* G. Forst. (HAMP20020)	Campanulaceae	Choak-a-thi	H	WP	Decoction	Tonsil (9), gallstone (16)	25	0.125	Not reported	Not reported
62	*Maesa indica* (Roxb.) A. DC. (HAMP20060)	Myrsinaceae	Arngeng	Sh	Lf	Paste	Toothache (4)	4	0.02	2 5 dihydroxy-6-methyl 3 (hemeos 16-enyl)-1 4benzoquinone [150]	Hypoglycemic, [151]
63	*Magnolia champaca* (L.) Baill. ex Pierre (HAMP20050)	Magnoliaceae	Ngiau	T	Lf	Miceration	Itchy eyes (18)	18	0.09	Butanoic acid, 2-methyl-3-oxo-, ethyl ester; Camphorsulfonic acid [152]	Anti-inflammatory, antipyretic, antimicrobial, anticancer [152, 153]
64	*Mangifera indica* L. (HAMP20002)	Anacardiaceae	Theihai	T	YS	Decoction	Diarrhoea (5), diabetes (10), hypertension (10), asthma (6)	31	0.155	Quercetin-3-O-β-L-rhamnopyranoside; maclurin-3-C-β-glucoside; amentoflavone; mangiferin [154]	Antidiabetic, antiviral, antiulcer, antimalarial, hepatoprotective, anti-inflammatory, antibacterial [155]
65	*Mentha arvensis* L. (HAMP20048)	Lamiaceae	Pudina	H	Lf	Decoction	Stomach problem (38)	38	0.19	Menthol, p-menthone, neo-menthone, iso-menthone [156]	Antioxidant, analgesic, antibacterial, cytotoxic, antifertility, anti-inflammatory, antiallergic [157]
66	*Mesua ferrea* L. (HAMP20022)	Clusiaceae	Herhse	T	Lf	Paste, decoction	Wound (8), stomachache (7), diarrhoea (3)	18	0.09	1,5-dihydroxyxanthone; euxanthone, 7-methyl ether; β-sitosterol [158]	Antimicrobial, anti-inflammatory, antiulcer, anti-arthritis, analgesic, analgesic, hepatoprotective, antioxidant [159]
67	*Mikania micrantha* Kunth. (HAMP20014)	Asteraceae	Japan Hlo	H, Cr	Lf	Paste, decoction	Wound (52), diarrhoea (8), dysentery (6), stomach ache (16)	82	0.41	β-cubebene; 1H-inden-1-one, 5-(1, 1-dimethylethyl)-2, 3-; allo-aromadendrene; β-caryophyllene [160]	Antimicrobial, antioxidant, anthelminthic [161, 162]
68	*Mimosa pudica* L. (HAMP20054)	Mimosaceae	Hlonuar	H	Lf	Decoction	Kidney disease (37)	37	0.185	7, 8, 3', 4'-tetrahydroxyl-6-C [alpha- L-rhamnopyranosyl-(1 – > 2)]-beta- D-glucopyranosyl flavone; catcher; 5, 7,3', 4'-tetrahydroxyl-6-C [alpha-L- rhamnopyranosyl-(1 – > 2)]-beta- D-glucopyranosyl flavone [163]	Wound healing, antimicrobial, analgesic, antimalaria, anti-inflammatory, hepatotoxicity, antidiarrhoeal, anthelminthes [164]
69	*Morus macroura* Miq. (HAMP20056)	Moraceae	Lung-li	T	Lf	Raw	Cut / wound (22)	2	0.01	Gallic acid, catechin, p-hydroxy benzoic, ellagic acid,3,4,5- methoxy cinnamic [165]	Anti-inflammatory, antioxidative, antiulcer [165]
70	*Musa acuminata* Colla (HAMP20059)	Musaceae	Balhla	H	Fr, sap	Paste, Decoction	Diabetic wound (6), lung disease (2), snake bite (4)	12	0.06	Pantothenic acid (B5), ferulic acid-hexoside, Vitamin C, provitamin A, lycopene [166]	Antioxidant, antidiarrhoea, immunomodulatory, anti-HIV, antidiabetic, hypolipidemic, anticancer, antimicrobial [167, 168]
71	*Mussaenda macrophylla* Wall. (HAMP20072)	Rubiaceae	Va-kep	Sh	Lf	Decoction	Internal bleeding (17)	17	0.085	3-O-β-d-glucopyranosyl-28-O-α-l-rhamnopyranosyl-16α-hydroxy-23-deoxyprotobassic acid; 16α-hydroxyprotobassic acid; 3-O-acetyldaturadiol; rotundic acid [169]	Antimicrobial [170]
72	*Ocimum americanum* L. (HAMP20049)	Lamiaceae	Runhmui	H	Lf, St	Paste	Pile problem (2)	2	0.01	á-pinene; farnesene; terpineol; farnesol; limonen [171]	Antioxidant, antimicrobial, [172, 173]
73	*Oroxylum indicum* (L.) Kurz (HAMP20018)	Bignoniaceae	Ar-chang- kawm	T	Br, Lf	Paste, decoction	Ulcer (6), arthritis (13), diarrhoea (3), dysentry (6), hepatitis C (9)	37	0.185	Ellagic acid; oroxylin-A; 7-o-methyl chrysin; palmitoleic; linoleic acids [174]	Anticancer, antimicrobial, hepatoprotective, antiulcer, amtiarthritic, anti-inflammatory, antioxidant, immunostimulatant, cardioprotective [175]
74	*Pandanus odorifer* (Forssk.) Kuntze (HAMP20064)	Pandanaceae	Ram-la-kuih	Sh	Lf	Decoction	Kidney disease 24	24	0.12	Not reported	Anticancer, antimicrobial [176]
75	*Parkia timoriana* (DC.) Merr. (HAMP20055)	Mimosaceae	Zawngtah	T	Fr, peel, Br	Paste, raw	Diabetes (10), baby umblical care (2), hypertension (4)	16	0.08	β-sitostero; javanicoside A; epigallocatechin gallate; ursolic acid; hyperin [177]	Antioxidant, antidiabetic, antiproliferative, antibacterial, anti-insecticidal [177]
76	*Phyllanthus emblica* L. (HAMP20065)	Phyllanthaceae	Sunhlu	T	Fr	Raw, juice	Diarrhoea (2), skincare (12), stomach problem (8), energybooster (7), diabetes (9), hypertension (6), jaundice (4)	48	0.24	Ascorbic acids, gallic acids, quercetin, punigluconin, emblicanin A&B, citric acids [178]	Antimicrobial, analgesic, antioxidant, antipyretic, hepatoprotective, antiulcer, antitumour, anti-inflammatory, immunostimulant [178]
77	*Picria fel-terrae* Lour. (HAMP20079)	Scrophulariaceae	Khatual	H	Lf	Decoction	Hypertension (24)	24	0.12	Rosmarinic acid; apigenin-7-O-β-D-glucuronide; picfeltarraegenin IV; acteoside; apigenin [179]	Antioxidant, anthelmintic, antiproliferative [180, 181]
78	*Plantago major* L. (HAMP20066)	Plantaginaceae	Kelba-an	H	Lf	Decoction	Kidney disease (2), urinary problem (34)	36	0.18	Plantaginin; plantagonin; ursolic acid; aucubin; palmitic acid, ascorbic acid [182]	Immunomodulator, antioxidant, analgesic, antiulcer, antibiotic, antiviral, cytotoxicity, hepatoprotective [182]
79	*Psidium guajava* L. (HAMP20061)	Myrtaceae	Kawl-thei	T	Lf	Decoction	Diarrhoea (97), hair fall (1)	98	0.49	β-sitosterol; guajanoic acid; oleanolic acid; uvaol; ursolic acid [183]	Anti-allergy, antioxidant, antimicrobial, anticough, antinogenetic, analgesic, cytotoxicity, antidiabetic, anti-inflammator [184]
80	*Pseudodrynaria coronans* (Wall. ex Mett.) Ching (HAMP20067)	Polypodiaceae	Awmvel	H	Lf	Paste	Herpes (12)	12	0.06	Kaempferol-3-O-(6'' -O-acetyl)-β-D-glucopyranoside; astragalin; isoquercitrin; kaempferol3-O-(6'' -O-feruloyl-4'' -O-acetyl)-β-D-glucopyranoside [185]	Antioxidant [185]
81	*Punica granatum* L. (HAMP20068)	Punicaceae	Thei-buh-fai	T	Fr	Raw, juice	Anemia (5), immuno- booster (21), cancer (5)	31	0.155	Ellagic acid; kaempferol; anthocyanins; punicalagin; quercetin; luteolin; ellagitannins [186]	Antiepileptic, antimicrobial, antiviral antidiabetic, antioxidant, anticancer, anti-inflammatory, hepatoprotective [186]
82	*Rhus chinensis* Mill. (HAMP20003)	Anacardiaceae	Khawmhma	T	Fr	Raw, powder	Diarrhoea (27), stomach ache (18)	45	0.225	Moronic acid; gallicin; 3-oxo-6β-hydroxyolean-12-en-28-oic; aciddimethyl caffic acid; phytol [187]	Antidiarrhoeal, anticancer, anti-HIV, antidiabetic, hepatoprotective [187]
83	*Sarcococca pruniformis* Lindl. (HAMP20040)	Euphorbiaceae	Pawhrual	H	Lf	Raw, decoction	Tonsil (49)	49	0.245	Not reported	Not reported
84	*Schima wallichii* Choisy (HAMP20083)	Theaceae	Khiang	T	Br/ St	Paste	Insect bite (9), wound (5)	14	0.07	Phenylpropanolamine; rotenone; glycidol; 2, 3-benzofurandione [188]	Anti-inflammatory, antioxidant, antimicrobial, analgesic, anti-pyretic [189, 190]
85	*Senecio scandens* Buch- Ham. ex D. Don (HAMP20015)	Asteraceae	Sai-ek-hlo	H, Cr	Lf	Decoction, paste	Ulcer (17), diabetes (13), Hypertension (8), toothache (3), wound (2)	43	0.215	Quercetin; kaempferol; luteolin; rutin; phytol, palmitic acid; β-Amyrin; β-Sitosterol [191]	Anti-inflammatory, analgesic, mutagenic, antimicrobial, antiviral, anti-tumour, anti-leptospirosis, antioxidant [191]
86	*Smilax glabra* Roxb. (HAMP20081)	Smilacaceae	Kai-tluang	H, Cr	Lf	Paste	Arthritis (20)	20	0.1	Palmitic acid; β-sitosterol; quercetin; apigenin; 3-methoxygallic acid; lignoceric acid [192]	Antimicrobial, antioxidant, cytotoxicity, anti-inflammatory, hepatoprotective, antiviral [192]
87	*Smilax perfoliata* Lour. (HAMP20080)	Smilacaceae	Kai-ha	Sh, Cr	Lf	Decoction	Dysentry (6)	(6)	0.03	Rutin,1; 6-O-diferuloyl-(3-O-p-coumaroyl)-b-D-fructofuranosyl-(2 → 1)-2-O-acetyl-a-D-glucopyranoside; cassiamin A & B; narcissin [193]	Antioxidant, antimicrobial [194]
88	*Solanum americanum* Mill. (HAMP20085)	Solanaceae	Anhling	Sh	Lf	Decoction	Nephrolithiasis (15), urinary retention (6), Kidney disease (11)	32	0.16	Pinoresinol; tetracosanoic acid; syringaresinol; β-sitosterol; scopoletin; medioresinol [195]	Antimicrobial, hepatoprotectivity, anti-inflammatory, anti-seizure, antioxidant, antipyretic, antitumour [196, 197]
89	*Solanum violaceum* Ortega (HAMP20086)	Solanaceae	Tawkte	Sh	Fr	Decoction, paste	Hypertension (20), diabetes (6), burn (4), boil (1), herpes (2)	33	0.165	7-oxositosterol; yamogenin; 7-oxostigmasteroldiosgenin [198]	Antioxidant, antiobesity. anthelminthic, cytotoxity, anti-inflammatory, analgesic, antinociceptive, antipyretic [199, 200]
90	*Spondias pinnata* (L. f.) Kurz (HAMP20004	Anacardiaceae	Tawi-taw	T	Br	Decoction	Diarrhoea (3)	3	0.015	Ellagitannins, galloylgeranin, lignoseric acid, β -carotein, oleanic acid [201]	Antimicrobial, cytoxicity, anti-cancer, antioxidant, anthelmintic, thrombolytic activity, antidiarrhoeal [201]
91	*Tectona grandis* L.f. (HAMP20089)	Verbenaceae	Teak	T	Br, Lf	Paste	Wound bleeding (2)	2	0.01	Gallic acid; β-sitosterol; betulinic acid; tectoquinone; squalene; lauric acid [202]	Antibacterial, anti-diabetic, antioxidant, antiulcer, antipyretic, anti-inflammatory, analgesic, antiviral, cytotoxic activity [202, 203]
92	*Zea mays* L. (HAMP20070)	Poaceae	Vaimim	H	Lf	Decoction	Kidney disease (15)	15	0.075	Eugenol; cis-α-terpineol; citronellol; 6,11-oxidoacor-4-ene [204]	Antimcrobial, antioxidant, antimutagen [205, 206]
93	*Zingiber officinale* Roscoe (HAMP20093)	Zingiberaceae	Sawhthing	H	Rh	Raw, decoction	Cold / cough (12), digestion (18)	30	0.15	Zingiberene; gingerols; farnesene; curcumene; zingerone; vitamins [207]	Appetiser, antimicrobial, immunostimulant, analgesic, anticancer antioxidant, antidiabetic, anti-inflammatory, antiarthritis, etc. [208]

^a^Habit: H, herbs; Sh, shrubs; Cr, creeper; T, tree; UR, used reports; FC, frequency of citation; UV, use value

^b^Part used, Lf, leaf; Br, bark; Fr, fruit, Rh, rhizome; St, stem; S, seed; WP, whole plants; YS, Young shoot

#### Plant use value

From the UV value evaluation, *Curcuma longa* L. (0.68), *Flueggea virosa* (Roxb. ex Willd.) Royle (0.63), *Psidium guajava* L. (0.49), *Chromolaena odorata* (L.) R.M. King & H. Rob. (0.43), *Mikania micrantha* Kunth. (0.41), *Citrus limon* (L.) Osbeck (0.34), *Carica papaya* L. (0.26), *Ananas comosus* (L.) Merr. (0.24), *Sarcococca pruniformis* Lindl. (0.24), *Phyllanthus emblica* L. (0.24), *Clerodendrum glandulosum* Lindl. (0.22), *Rhus chinensis* Mill. (0.22), *Senecio scandens* Buch- Ham. ex D. Don (0.21) were reported to have the highest use value (UV).

#### Informant consensus factor

We calculated the informant consensus factor by categorising the reported illness into 16 ailment groups along with the number of users report and taxa (Table 5). In our study, *F*_ic_ values ranged from 0.866 to 0.962 which were all close to 1.

**Table 5:** Informant consensus factor with their used report in each of an ailment category

Illness categories	No. of used report	No of taxa	*F*_ic_
Dental Care (DC)	57	8	0.875
Skin Care (SC)	259	13	0.953
Hair Care (HC)	16	3	0.866
Eyes/Nose/ Ears/Mouth (ENT)	159	9	0.949
Genito-urinary Disease (GUD)	139	9	0.942
Kidney Disease (KD)	196	15	0.928
Endocrinal Disorder (ED)	175	12	0.936
Cancer Disease (CD)	75	5	0.945
Liver Problem (LP)	100	7	0.939
Cardiovascular Problem (CP)	222	14	0.941
Muscle/Bone Problem (MBP)	81	4	0.962
Respiratory System illness (RSI)	42	5	0.902
Gastro-intestinal Disease (GID)	940	42	0.956
Wound healing (WH)	126	10	0.928
Poisonous Bites (PB)	45	5	0.909
General Health (GH)	81	10	0.887
	2717		

#### Relative importance and cultural value

Results of top-ranking species in terms of both indices of relative importance and cultural value are given in Table 6. This study elucidates the highest cultural valued species and relative importance species utilised by the inhabitants of the study area. In general, the evaluated values were quite high in case of CVs and an average value of RI (0.607 ± 0.38) clarified that the versatile species, i.e. *Phyllanthus emblica* (RI = 2) was 3.3 times more relevant than the rest of the listed species.

**Table 6:** Species with high cultural values and relative importance

CVs	RI
*Curcuma longa* (27.28)	*Phyllanthus emblica* (2)
*Flueggea virosa* (16.0)	*Carica papya* (1.85)
*Embelia vestita (*13.12)	*Senecio scandens* (1.54)
*Psidium guajava* (11.64)	*Ananas comosus* (1.54)
*Citrus limon* (7.16)	*Oroxylum indicum* (1.38)
*Mikania micrantha* (6.72)	*Chromolaena odorata* (1.37)
*Chromolaena odorata* (3.40)	*Allium sativum* (1.23)
*Euphorbia milii* (3.17)	*Ensete superbum (*1.23)
*Sarcococca pruniformis* (2.82)	*Centella asiatica* (1.23)
*Clerodendrum glandulosum* (1.93)	*Measia indica* (1.23)
*Rhus chinensis* (1.82)	*Solanum violaceum* (1.21)
*Mimosa pudica* (1.129)	*Capsicum annuum* (1.07)

CVs, caltural value; RI, relative importance

#### Correlation and validation studies

An attempt was made to compare the use of all the medicinal plants reported by the informants with the previous papers published for their biological activity or ethnomedicines (Table 4). According to the studies conducted by Cakilcioglu et al., 2011, it was stated that if a medicinal plant has been reported for similar use in other parts of the world, its pharmacological effect could be more easily known [209].

The use of crude juice of *Allium cepa* L. showed a significantly higher hair growth rate than tap water when applied twice a day for two months which corroborated the present report of hair regrowth [22]. Metallothionein, an antioxidant protein present in *Aloe vera* (L.) gel, has been reported to have a protective effect against UV and gamma radiation damage to the skin. It scavenges free radicals by preventing the suppression of glutathione peroxidase and superoxide dismutase in the skin [30]. So, this validated the used of *A. vera* for skin care and burning by the Mizo tribes. In the present study, *Betula alnoides* Buch- Ham. ex D. Don has been used as toothpaste for whitening teeth while it was proved that 80% methanolic bark extract had the potential α-glucosidase inhibitory effect that prevent the (98.4%) at 40 µg/mL concentration [2]. *Cajanus cajan* (L.) Millsp is used effectively in Champhai district to treat jaundice and intestinal worms. To certify this, the methanolic extracts showed hepatoprotective activity in Swiss albino mice by inducing carbon tetrachloride (CCl_4_) that cause liver damage. It lowers the serum levels of glutamate pyruvate transaminase (SGPT), or alanine aminotransferase (ALT) aspartate aminotransferase (AST) or serum glutamate oxaloacetate transaminase (SGOT) significantly [58].

When the aqueous extracts of *Carica papaya* L. and *Ananas comosus* L. were given to Spraque Dawley rats orally at doses of 5 and 10 mg/kg, both possessed mild to strong diuretic activity. Careful measure should be taken when using these plants as increase in the level of urinary K^+^, serum BUN and creatinine were mentioned [71]. This validated the used of *C. papaya* and *A. comosus* in kidney disease and urinary infection. The contemporary reports showed that *Drymaria cordata* (L.) was used as an instant pain killer for rheumatism; meanwhile, the scientific study also demonstrated that the aqueous whole plant extract exhibited analgesic and antipyretic properties at doses of 100, 200, and 400 mg/kg p.o mediated through peripheral and central mechanisms [210]. The latex water-soluble fraction of *Euphorbia royleana* Boiss. showed dose-dependent anti-arthritic and anti-inflammatory effects in rats and mice administered through gavage at doses of 50–200 mg/kg having more than 1500 mg/kg oral LD_50_ in both [135]. Dose-dependent and significant decline in the number of abdominal constrictions induced by intraperitoneal administration of acetic acid was observed in methanol extract of *Lablab purpureus* (L.) Sweet. at a dose of 200 mg and 400 mg exhibited far better analgesic activity than 200 mg aspirin per kg of body weight [211].

*Colocasia esculenta* (L.) Schott and *Elaeagnus caudata* Schltdl. ex Momiy. were declared to use to discharge placenta after birth and to treat vaginal discharge (Lochia) for women in present study. Besides this record, in Cachar hills district of Assam, India, 5 ml of *Elaeagnus caudata* fresh root extract diluted in 10 ml of fresh water was also administered orally once a week to prevent miscarriage during pregnancy although there is no scientific study to backup this claim [118]. Apart from present report in Jamaica, *Mikania micrantha* Kunth. was most popularly used too for wound healing and its extract showed anti-inflammatory and antimicrobial activity against common pathogens, namely *Escherichia coli*, *Staphylococcus aureus* and *Streptococcus* sp. [212]. The decoction of *Psidium guajava* leaf was effectively used for diarrhoea which already proved to have antidiarrhoeal and protein conservative effects in diarrhoeal rats at a dose of 50 and 100 mg/kg of body weight. It increased the kidney weight and concentration of sodium, potassium and chloride significantly [213]. In the animal study of anti-urolithialic activity of *Solanum nigrum*, the fruit hydroalcoholic extract elicited potent activity against calcium oxalate urolithiasis effected by ethylene glycol through tumour necrosis factor adiponectin stimulation and alpha inhibition, also maintained the balance between stone promoter and inhibitor such as calcium and magnesium, respectively [214]. Thus, this authenticated the used of *S. nigrum* for removing kidney stone by the Mizo tribes in India.

*Anoectochilus brevilabris* Lindl.*, Begonia inflata* C.B. Clark*, Dysoxylum excelsum* Blume*, Embelia vestita* Roxb*, Ensete glaucum* (Roxb.) Cheesman*, Gomphogyne cissiformis* Griff.*, Helicia robusta* (Roxb.) R. Br. ex Blume*, Laurocerasus undulata* (Buch- Ham. ex D. Don) M. Roem. and *Lobelia angulata* G. Forst., *Sarcococca pruniformis* Lindl. were the plants that did not have biological activity reported previously which means that there is no scientific validation to support their application. Therefore, these plants were especially recommended in carrying out further investigation.

In addition, we compiled the secondary metabolite isolated chemical constituents done by several researchers for all the documented plants in the present study. Further investigation revealed that secondary metabolites from 13 plant species that have neither less nor none chemical compound isolated or identified—*Anoectochilus brevilabris* Lindl., *Begonia inflata* C. B. Clark., *Castanopsis tribuloides* (Sm.) A. DC., *Combretum wallichii* DC, *Elaeagnus caudata* Schltdl. ex Momiy., *Embelia vestita* Roxb., *Ensete glaucum* (Roxb.) Cheesman, *Gomphogyne cissiformis* Griff., *Helicia robusta* (Roxb.) R. Br. ex Blume, *Laurocerasus undulata* (Buch- Ham. ex D. Don) M. Roem., *Lobelia angulata* G. Forst., *Pandanus odorifer* (Forssk.) Kuntze, *Sarcococca pruniformis* Lindl. (Table 4) which will surely have great potent on ethnopharmacological study.

## Discussion

According to our findings, women practitioners (44%) were less than men (56%) which may be explained partly by the low sex ratio of the district; however, it can be assumed that women play lesser role in ethnomedicinal practices [215, 216]. Among self-employed, farmers account for 58.5%, business persons 34.2% and carpenters were 21.4%. Farmers represented the highest percentage as they often lack access to modern healthcare facilities due to various issues ranging from financial, transportation and higher education. These issues forced them to rely on traditional medicines, cultivating and utilising them more regularly than others and somehow playing a big role in conservation too. Through this study, we observed that young informants like students around 18 to 25 years old have little expertise in practicing ethnomedicine and utilised them rarely as compared to elder informants. This may be due to change in mentality brought by education to rely only on prescribed medicines. Further, the results of the usage of plants dominated by the families were followed and confirmed the work done by some researchers stating that greater the plants grew in the study area the more it will be favourably and commonly used [217]. This supports the non-random plant selection hypothesis by Moerman 1979 [218]. Large families such as Asteraceae and Euphorbiaceae were most utilised while Orchidaceae and Poaceae were underutilised (low used report). However, due to non-random selection, small families like Cucurbitaceae and Zingiberaceae became over-represented (high used report). Thus, this implies that medicinal plants are not selected randomly by the inhabitants of Champhai district but are utilised based on their cultural and traditional knowledge [219]. In the present study, we laid out the only accepted botanical names by ‘The Plant List’ and their family, local name, habit, mode of preparation and ailments as illustrated in Table 4.

Out of 93 species, 40 were cultivated species, whereas 53 were found in the wild. There were also 6 invasive alien species most notably *Chromolaena odorata* and *Mikania micrantha* which were commonly used to treat wounds topically. This is because wounds are the most common form of injury and these two species can be found almost everywhere [219]. The frequent use of herbaceous plants as medicines among the informants was due to their richness, abundance as well as their ability to grow easily in nature. Meanwhile, many parts of the world have been commonly using herbs as their medicinal ingredients due to their wide range of medicinal properties [220]. Leaves are the most utilised part of the plants due to their ease off collection as compared to their underground part. It is also the active site of photosynthesis accompanied by the production of metabolites [1, 221]. In addition, leaves can be easily prepared and stored. It can be dried quickly under the sun in lesser amount of time than other parts like stem, bark and rhizome.

Similarly, it is also reported that decoction was the most common preparation method for herbal medicine while in some other tribal community [3], preparation of paste was the most common method applied [1, 216, 222]. For decoction the plant part was washed thoroughly and boiled with water administering the juice orally, whereas for paste the materials were crushed or rubbed within palms and applied topically. To make fine powder plant parts were shade dried and ground. Intake of oral administration and external topical formulation were the main mode of administration used in traditional herbal medicines which has also been previously reported [215, 223]. Regarding the duration of consumption of herbal medicine, it depends on the illness whether it was short term or long term. For instance, short-term illness like cold, flu, stomach upset and skin problem, the consumption period did not last more than 1 week. On the contrary, the long-term illness like diabetes, kidney failure and heart diseases, the consumption period of plants (e.g., *Flueggea virosa*) was much longer and last more than a month and so on.

The inhabitants of the study area extensively exploited medicinal plants to treat various illnesses and other needs which have not been previously reported. For instance, *Anoetochilus brevilabris* was used for pile treatments, *Betula alnoides* as toothpaste, *Capsicum annuum* to soothe and prevent scars from skin burns. *Colocasia esculenta* to expel lochia, *Euphorbia milii* as antidiarrhoea, *Lablab purpureus* as a pain reliever, *Mussaenda macrophylla* to stop internal bleeding and *Parkia timoriana* for treating baby umbilical cord. From this study it was clear that among the informants, stomach problems like ulcer, indigestion, diabetes, hypertension and kidney problems were common illness resulting in high user rate of consuming herbal medicines and similar record was reported by Mahwasane et al., [224]. Further, skin problem like dermatitis which was the second highest usage report was the highest ailment in most other tribal communities like Malda district in West Bengal reported by Saha et al. [225].

Generally, majority of the informants did not consume the medicines prescribed by the Doctor’s prescribed medicines along with their herbal medicine and claimed that many plants like *Sarcococca pruniformis* (tonsil), *Psidium guajava* (diarrhoea), *Mikania micrantha* (cut/wound), *Flueggea virosa* (chicken pox), *Elaeagnus caudata* (veginal discharge) were really effective and most importantly, none of them reported any adverse effect such as vomiting, headache, nausea, allergic reactions and/or skin rashes. Moreover, regarding the expenditure on buying medicines, 38% of the informants usually purchased their herbal medicines either in raw form (*Allium sativum, Allium cepa, Beta vulgaris*) or in processed form like juice (*Citrus limon, Phyllanthus emblica, Citrus aurantiifolia*), fruits (*Punica granatum, Phyllanthus emblica, Cucumis sativus*), and powder (*Curcuma longa*). Concerned about the source of their knowledge, all the informants reported that they have heard and learned some of their information from their elders, family and/or acquaintances. Besides these, 30% of the informants have also gathered additional information through social media and 10% through books, magazines and newspapers. This documentation clearly showed that knowledge and cultural practices of herbal medicines had been shared through the indigenous community through word of mouth.

Frequency of citation showed the sociocultural importance of medicinal plants to identify their therapeutic value [16]. The FC value is directly proportional to the use value (UV), the more FC value will increase the used value significantly.

*Curcuma longa* L. is one of the main commercially grown as seasoning plants in India. In Southeast Asia including India and China, turmeric powder has been used extensively for spice and colouring food material. It had a wide range of medicinal value that curcumin was the main bioactive chemical constituents [226]. *C. longa* was a mandatory spice that each and every household kept it that’s why the reason used report (UR) for medicinal value and cultural value (CVs) were high among the informants. In case of CVs, the high value was due to a greater number of the used report with lesser-used categories. The informants in present study reported that *Flueggea virosa* have a prominent effectiveness against diabetes (59 UR) and chickenpox (50 UR). The Mizo tribes extensively used *F. virosa* and *Embelia vestita* Roxb. plant to treat chickenpox and measles by bathing once a day with the decoction of leaf mixed with water. Apart from the degree of the used report, this index also attributed to the effectiveness of their use and importance.

Higher in the UV value indicates the more rate of agreeing and sharing their knowledges and practices of the medicinal plants among the informants [216]. Among the Terai forest of western Nepal *Curcuma longa* L. was also reported as the highest used value [227] similar to this result.

The plants with low UV value were *Colocasia esculenta* (L.) Schott, *Eulophia nuda* Lindl. and *Ocimum americanum* L., *Maesa indica* (Roxb.) A. DC, *Morus macroura* Miq, *Tectona grandis* L.f., *Hibiscus sinensis* Mill, *Elaeis guineensis* Jacq, *Smilax perfoliata* Lour with less than 0.05 UV as shown in Table 4. *Tectona grandis* L.f. was also described with very low UV value by Ayyanar and Ignacimuthu as relevant to this result [1]. According to Chaudhary et al. 2006, the plants with low used value were in at risk of misrecollecting and passing on to the young generation which might be gradually disappearing [228]. On the other hand, the relevance of knowing the plant used value was for the convenience of pharmacological study and their used reliability [229].

However, Rajakumar and Shivanna had mentioned that the value of *F*_ic_ depends on the accessibility of the taxa for the treatment of various diseases in the study area. Muscle/Bone problem with 81 UR have the highest *F*_ic_ value of 0.962 followed by gastro-intestinal disease (GID) with 940 UR and skin care (SC) with 259 UR (Table 5). The lowest *F*_ic_ value in the present study was the General Health (GH) category (Cold, fever, immunity boost) with 0.887 which was still more than the previous maximum *F*_ic_ value report in Shimoga district, Karnataka, India i.e. 0.77 in Liver complaints [230]. Most RI value (*Phyllanthus emblica*) is considered to be versatile on its uses which would also increase the importance of the plant when it is used to treat more illnesses. The high RI values of some species may be attributed to their abundance and availability in the study area [19].

Overall, the quantitative analysis revealed that *Curcuma longa* was the most relevant species with the highest used value, frequency of citation and cultural value except in relative importance. This is due to the fact that the RI value is independent of the number of informants used report. On the conflict of these report, our study indicated that there was high consistency of the indigenous informant knowledge in the practices of ethnomedicines and utilised the same plants to treat it.

## Conclusions

The present study concluded that the native people in the study area have their unique way of utilising medicinal plants to treat different kinds of ailments. We documented 93 valuable medicinal plants belonging to 55 families and 85 genera in which Euphorbiaceae and Asteraceae family were the most widely used in the area. This study supported the non-random selection of medicinal plants hypothesis. Among the plants part, leaves were the most commonly used. No new medicinal taxa were reported, but this study is a first quantitative report of ethnomedicine in this region and no informant had reported an adverse effect of herbal medicines. Their traditional pieces of knowledge had been passed on from their elders mostly through word of mouth. This study also revealed that younger generations between the ages of 18 and 30 have little to no knowledge of preparation of herbal medicines and their use as compared to the older age groups. This is mostly due to the availability of modern clinical drugs in the villages. Therefore, the traditional knowledge and practices of medicinal plants in the study area are somehow at risk of dying. This is why it is important to document the valuable knowledge as well as for conservation of the taxa.

The use of quantitative indices was essential in the field of ethnobotany to determine the most valuable plants along with their role played in a particular culture and to develop conservation initiatives. The plants which have high usage report and frequency of citation were known to possess numerous phytochemical compounds. The calculated informant consensus factor was extremely high, which means that the acquired data can be used as reference and reliable for ethnopharmacological study in the future. Even though the remedial value of many high cited plants has already been verified, there are still some plants that need to be validated. Hence, they are strongly recommended for further studies to develop alternative drugs.

## Data Availability

All data generated or analysed during this study are included in this published article.

## Abbreviations

H: Herbs
Sh: Shurbs
Cr: Creeper
T: Tree
UR: Used reports
FC: Frequency of citation
UV: Use value
Lf: Leaf
Br: Bark
Fr: Fruit
Rh: Rhizome
St: Stem
S: Seed
WP: Whole plants
YS: Young shoot
